# Researching Complex and Multi-Level Workplace Factors Affecting Disability and Prolonged Sickness Absence

**DOI:** 10.1007/s10926-016-9660-3

**Published:** 2016-08-22

**Authors:** Vicki L. Kristman, William S. Shaw, Cécile R. L. Boot, George L. Delclos, Michael J. Sullivan, Mark G. Ehrhart, the Hopkinton Conference Working Group on Workplace Disability Prevention, Benjamin C. Amick, Johannes R. Anema, Elyssa Besen, Peter Blanck, Cécile R. L. Boot, Ute Bültmann, Chetwyn C. H. Chan, George L. Delclos, Kerstin Ekberg, Mark G. Ehrhart, Jean Baptiste Fassier, Michael Feuerstein, David Gimeno, Vicki L. Kristman, Steven J. Linton, Chris J. Main, Fehmidah Munir, Michael K. Nicholas, Glenn Pransky, William S. Shaw, Michael J. Sullivan, Lois E. Tetrick, Torill H. Tveito, Eira Viikari-Juntura, Kelly Williams-Whitt, Amanda E. Young

**Affiliations:** 1 Department of Health Sciences, Lakehead University, 955 Oliver Road, Thunder Bay, ON P7B 5E1 Canada; 2 Institute for Work and Health, Toronto, ON Canada; 3 Division of Human Sciences, Northern Ontario School of Medicine, Lakehead University, Thunder Bay, ON Canada; 4 Dalla Lana School of Public Health, University of Toronto, Toronto, ON Canada; 5 Liberty Mutual Research Institute for Safety, Hopkinton, MA USA; 6 University of Massachusetts Medical School, Worcester, MA USA; 7 Department of Public and Occupational Health, EMGO Institute for Health and Care Research, VU University Medical Center, Amsterdam, The Netherlands; 8 Division of Epidemiology, Human Genetics, and Environmental Sciences, The University of Texas-Houston School of Public Health, Houston, TX USA; 9 Center for Research in Occupational Health (CiSAL), Pompeu Fabra University, Barcelona, Spain; 10 McGill University, Montreal, QC Canada; 11 Department of Psychology, San Diego State University, San Diego, CA USA

**Keywords:** Employer practices, Workplace factors, Research priorities, Disability management

## Abstract

*Purpose* There is growing research evidence that workplace factors influence disability outcomes, but these variables reflect a variety of stakeholder perspectives, measurement tools, and methodologies. The goal of this article is to summarize existing research of workplace factors in relation to disability, compare this with employer discourse in the grey literature, and recommend future research priorities. *Methods* The authors participated in a year-long collaboration that ultimately led to an invited 3-day conference, “*Improving Research of Employer Practices to Prevent Disability,* held October 14–16, 2015, in Hopkinton, Massachusetts, USA. The collaboration included a topical review of the literature, group conference calls to identify key areas and challenges, drafting of initial documents, review of industry publications, and a conference presentation that included feedback from peer researchers and a question/answer session with a special panel of knowledge experts with direct employer experience. *Results* Predominant factors in the scientific literature were categorized as physical or psychosocial job demands, work organization and support, and workplace beliefs and attitudes. Employees experiencing musculoskeletal disorders in large organizations were the most frequently studied population. Research varied with respect to the basic unit of assessment (e.g., worker, supervisor, policy level) and whether assessments should be based on worker perceptions, written policies, or observable practices. The grey literature suggested that employers focus primarily on defining roles and responsibilities, standardizing management tools and procedures, being prompt and proactive, and attending to the individualized needs of workers. Industry publications reflected a high reliance of employers on a strict biomedical model in contrast to the more psychosocial framework that appears to guide research designs. *Conclusion* Assessing workplace factors at multiple levels, within small and medium-sized organizations, and at a more granular level may help to clarify generalizable concepts of organizational support that can be translated to specific employer strategies involving personnel, tools, and practices.

## Introduction

The fundamental responsibility of employers to help workers who are ill or injured to stay on the job has been a longstanding principle in the creation of business laws, regulations, and best practice guidelines to protect workers since the time of the early twentieth century industrial revolution [1, 2]. Accordingly, most accumulated research in occupational health and safety has focused on the primary prevention of illness, injury, and hazardous exposures in the workplace. A more recent body of evidence has shown that workplace factors not only correlate with injury rates, but also with disability duration for those workers who become ill, injured, or physically or mentally impaired [3]. This evidence, coupled with the vested interest of employers and insurers to reduce unnecessary disability costs, has supported a growing interest in proactive return-to-work (RTW) and disability management (DM) practices in the workplace. Providing modified duty and other formal accommodations is a key factor, but other characteristics of work and the work environment have been correlated with sickness absence, disability claim duration, and perceived work ability. More research is needed to assess and interpret the workplace factors consistently associated with risk of long-term sickness absence or permanent disability.

*Workplace factors*, in the context of this article, refers to variables that have been measured or assessed at the workplace or organizational level as potential correlates with long-term sickness absence and work disability. These factors need to be distinguished from individual level psychosocial factors that refer to psychological, social, and environmental factors that have also been shown to impact recovery, progression, and recuperation from illness and disease [4]. Workplace issues surrounding work disability can be viewed from a number of perspectives, including workers, healthcare practitioners, employers, insurers, and researchers [3]. There is growing research evidence that workplace factors influence disability outcomes, but these variables have been assessed with different stakeholder perspectives, measurement tools, and methodologies.

With a goal toward improving future research of employer disability prevention strategies, the authors participated in an invited 3-day conference, “*Improving Research of Employer Practices to Prevent Disability,* held October 14–16, 2015, in Hopkinton, Massachusetts, USA. Methods and general proceedings of the conference are described in the introductory article to this special issue [5]. The authors of the present article represented a sub-group tasked with understanding the state of the science with regard to workplace factors and their effect on disability outcomes. We were asked to review the applicable scientific literature, assess its relevance for employer disability management strategies, compare factors described in the scientific and employer-directed grey literature, contrast key conceptual and theoretical frameworks, and recommend future research priorities.

In this paper, we first briefly review workplace factors identified from the published peer-review literature. In addition to highlighting the important factors, we also discuss typical research methodologies, assessment domains, and conceptual frameworks or theoretical models that have guided this research. Second, we review the 33 employer-directed publications (“grey literature”) made available by the conference organizers to examine the employer perspective on important workplace factors and models that employers use to determine these factors. Next, we examine the disparities between factors identified through research and those that appear prominent in employer discourse. In the absence of a unified conceptual framework, we propose three basic principles as a building block towards the development of a conceptual framework. Finally, we conclude with a review of existing research limitations and recommendations for future research on workplace factors associated with work disability.

## Typical Research Methodologies

Historically, an epidemiological approach has been the most common methodological approach towards research on workplace factors associated with work disability. Research designs commonly used in this field include case series, cross-sectional studies, case–control designs, and cohort studies. Cohort studies and secondary analyses of randomized controlled trials have examined disability prognosis [6, 7].

More recently qualitative and mixed-methods designs have become popular [8, 9]. This may be due to the difficulty of obtaining sufficient sample sizes to appropriately power quantitative studies. Also, the types of research questions being asked are more suited to qualitative methods. Participatory action research (PAR) has started to play a prominent role [10], especially in the area of participatory ergonomics [11–15]. With PAR, researchers and participants work collectively to identify problems, resources, and sustainable solutions. PAR strives for understanding through collaborative change and reflection. It emphasizes collective inquiry and experimentation grounded in experience and social history. Newer statistical approaches that take into account some of the complex interrelationships between contextual (e.g., workplace) and individual (e.g., worker) variables are being applied to the analysis of disability outcomes. Among these are multi-level analyses [16], latent trajectory analysis [17, 18] and frailty models [19].

We restricted our summary of the literature to those studies with existing workers; that is, individuals with health problems who are still tied to identifiable employers. Other related bodies of scientific literature have focused on hiring practices and job search strategies for individuals with disabilities, also on ways to return permanently disabled workers back to the competitive job market. These were outside of our scope as defined by the organizing committee for the conference, but workplace factors are certainly relevant issues for these topics as well.

## Predominant Workplace Factors

A number of systematic reviews have identified several workplace factors associated with work disability [3, 20]. For summary purposes these factors can be divided into four categories: (1) Physical job demands, (2) Psychosocial job demands, (3) Work organization and support, and (4) Workplace beliefs and attitudes.

Physical job demands include high pace of work, blue- versus white-collar workers, job difficulty, vibration, awkward postures, construction industry, self-reported high physical work, and objectives measures of high physical work [21–34]. There is strong evidence for high physical job demands to be positively associated with work disability [3, 20]. Physical job demands are most often self-reported by the worker [3].

Psychosocial job demands include lack of job control, short job tenure, high job stress, high job demands, low fairness and distributive justice, and role ambiguity [6, 21, 24–27, 29, 30, 35–42]. Strong evidence is available for job strain, increased psychological demands, and lack of worker control; but only moderate evidence for lack of job control and fairness [20].

Work organization and support factors include low social support from colleagues and supervisors, few offers of job modification, limited accessibility, part-time work, low leadership quality within the workplace, and little managerial involvement [6, 8, 9, 21, 23–25, 27, 28, 30, 31, 34, 35, 38, 40, 42–48]. Systematic reviews have identified strong evidence for lack of social and supervisory support and moderate evidence for part-time work, poor leadership quality, and lack of managerial involvement [20].

Workplace beliefs and attitudes include low job satisfaction, negative feelings towards work, low occupational pride, and trouble at work [26, 27, 30, 35, 38, 45, 49]. Although there is a strong evidence base for low job satisfaction [20], the association between workplace beliefs and attitudes and work disability may be more complex than what can be captured in a job satisfaction variable [3].

## Typical Health Issues Studied

Research on workplace factors has primarily focussed on musculoskeletal disorders (MSK), predominantly back pain. Back pain has most often been studied in the field of work disability, in particular in the US and Canada. In Europe, and more recently in Canada, a larger focus on mental health has developed, which can be explained partly by differences in jurisdictions.

The focus on MSK and back pain can be explained by the relevance of both categories for receiving benefits in US and Canada, where only workers with work-related sick leave are entitled to benefits. In Europe, (e.g., in the Netherlands) every employee receives full salary for 1 year in case of sick leave, regardless of the cause. In recent years, mental health issues are gaining attention from researchers as this is becoming the primary cause for work disability in Europe. In the area of mental health problems, a number of recent reviews have been published on bullying and aggression as important workplace factors to cause mental health problems [50–52]. Research on MSK health focuses on different variables compared to mental health, as different conceptual models are applied. For example, the ergonomics framework for MSK health or the job demand resources model for mental health. Related to these models, different variables are included in studies on workplace factors, such as a focus on ergonomic variables related to work station design, or psychosocial workplace factors related to the job demands resources model.

## Emerging Research Topics

### Cancer

As cancer treatments improve, workplace issues for cancer survivors are becoming more important [25, 30]. Supervisor support and type of occupational setting are important factors. A more recent review concluded that focussing on work-related goals rather than on return to work would be beneficial for cancer survivors [53]. In general, more recent work on cancer survivors focuses on individual–level factors rather than workplace factors.

### Other Chronic Conditions

Some reviews focus on specific chronic conditions, such as spinal cord injury [40, 54], stroke [28, 55], and traumatic brain injury [56]. Substantial overlap exists between workplace factors associated with disability across the assessment domains. Some workplace issues may have differing effects on various conditions depending on the nature of the underlying medical problem. For example, heavy physical work may have more significant impacts for an individual with back pain than for someone recovering from depression, but there are few studies testing such condition-specific interactions [3]. This is likely due to the low prevalence of many of these conditions and the number of different work settings, which make it difficult to study a specific workplace factor. In line with the ICF Model, it might be of added value to study different chronic conditions in one study, as personal and environmental factors might have a similar influence on work participation in different chronic conditions. In addition, the difficulties in research related to comorbidity and multi-comorbidity may be solved by taking a generic approach to chronic conditions rather than focusing on a specific condition.

### Aggregated Analysis of Sickness Absence Across Multiple Health Conditions

White and colleagues recently conducted a review on workplace factors contributing to sickness absence across different health conditions [20]. They concluded that lack of social support, increased physical or psychological demands at work, job strain, lack of supervisory support, low job satisfaction, low job control, and poor leadership quality were significant predictors of sickness absence for at least two different health conditions [20]. These findings support an approach towards investigating beyond specific diagnosis, therewith creating opportunities for collaboration, and joining forces of different research groups.

### Workplace Aggression and Bullying

Over the past 5 years, 4 reviews have been published on bullying and aggression at the workplace. This topic is getting increased attention as it is associated with a large psychological impact [51] and may lead to both mental and somatic health problems [52]. Recent studies suggest that more than half of US organisations are affected by aggression [57]. Aggression may occur in worker-client (patient, customer) relationships, but also in worker–worker interaction and may range from verbal to physical abuse [51]. Workers with disabilities and frequent sickness absence may be at greater risk of workplace aggression and bullying [58] and this may be a possible factor in long-term work outcomes.

## Conceptual Frameworks Guiding Research

Various conceptual frameworks have been used to describe work disability prevention in the research literature and to identify possible workplace factors. A few of these models and examples of their use are described in Table 1. There is no single parsimonious multi-variable model that can explain the role of workplace factors in occupational disability. While this would be beneficial, it may take some time to come to fruition. Such a model that addresses only workplace factors will have limited explanatory power. Many of the factors that might contribute to delayed workplace re-integration are likely to be affecting some workers more than others. Characteristics of workers likely act as ‘moderators’ of the impact of workplace characteristics. We advocate for starting with the most basic principles as the building blocks of a conceptual framework and discuss this later under “Implied or actual theoretical perspectives guiding research and practice”.

**Table 1:** Work disability research models

Conceptual model	Model features	Example studies
Biomedical model [60]	Defines disability in terms of the extent of impairment or degree of handicap as well as the clinical response. According to this model, work disability is explained by the severity of the condition, the effectiveness of clinical treatment, the strength of economic disincentives, and the effectiveness of the employer’s disability management approach [60]. Few workplace factors are considered here beyond economic (dis)incentives to return to work	Work injury compensation and the duration of non-work spells [61]
Biopsychosocial model [62]	This model highlights health and illness as the product of a combination of factors, including biology, behavioural factors, and social conditions, yet the workplace is still not specifically included	Predicting non return to work after orthopaedic trauma: the Wallis Occupational Rehabilitation Risk (WORRK) Model [63]
International Classification of Functioning (ICF) [64]	Describes disability as a matter of how the person responds to life activities and social participation in the presence of contextual factors [64]. Yet, there seems little research available using the ICF model as a framework for research on work disability [65]	Predictive factors of work disability in rheumatoid arthritis: a systematic literature review [23]
Karasek job demand-control model (JDC) [66]	This model provides a mechanism for predicting work stress when the work tasks are too burdensome [66]. The JDC model assumes that employee health and work motivations are explained by two characteristics of the work situation: work demands, which include working quickly and having sufficient time to complete the work; and control over how to perform the work [67]. The premise for the model is that high demands can lead to high job strain, but can be moderated by high job control. Social support has also been found to moderate the effects of high job strain [68]. Many physical, psychosocial, and work support factors have been identified and tested for their effects on work disability through the use of this model	The demand-control-support model as a predictor of return to work [69]
Feuerstein model [70] and Institute of Medicine (IOM) [71]	This model for work re-entry of people with upper extremity musculoskeletal problems was the first work disability model to specifically include workplace factors [70]. This model is based on musculoskeletal injury causation and behavioural research and demonstrates that return to work is a result of interactions between behaviour, medical status, physical capabilities, and work demands. Similarly, the Institute of Medicine (IOM) model indicates the complex linkages among the worker’s biology, psychology, workplace, and work disability [71]. Both of these models include workplace, and not just worker, factors in the disability problem and have led to the study of multiple psychosocial, behavioural and work organization factors [72–74]	Clinical and workplace factors associated with a return to modified duty in work-related upper extremity disorders [75]
Effort-Reward Imbalance (ERI) model [76]	This model predicts health based on psychosocial occupational stress [76]. In the model, stress is an outcome of an imbalance between the efforts paid by the employee (job demands, obligations, critical coping, and need for control) and the rewards received from the employer and society (money, esteem, status, job security) [67]. Workplace factors such as psychosocial job demands, work organization, and workplace attitudes have been identified and studied through the ERI model [77–79]	Effort-reward imbalance as a risk factor for disability pension: the Finnish Public Sector Study [77] Effort-reward imbalance at work and general health of Las Vegas hotel room cleaners [78]
Case-management ecological model [80]	This model provides an operational paradigm to guide case-management operations or to detect various systems on the disability process [80]. It was not developed to explain the factors leading to work disability, but rather to identify the systems and stakeholders involved in the work disability process. It provides an opportunity to identify actors and variables from various levels within the four systems of the work disability arena: personal, legislative and insurance, workplace, and healthcare	Management of return-to-work programs for workers with musculoskeletal disorders: a qualitative study in three Canadian provinces [81]
Job Demands-Resources model [82]	This recent model has been used to confirm sickness absence [83, 84]. Job demands refer to the physical, social, and organizational aspects that require physical or psychological efforts. Job resources refer to the physical, psychological, social, or organizational aspects of the job that reduce demands [82]. This model has been used to demonstrate burnout and subsequently sickness absence [84]	How changes in job demands and resources predict burnout, work engagement, and sickness absenteeism [84]
Faucett’s integrated model [85]	This model distinguishes between external workplace factors and individual level factors. Work environment factors include functional—job-specific factors, temporal—timing of work factors, physical—biomechanical ergonomics, and interpersonal—social factors such as solitary work or supervision. Most studies using this model have examined development of work-related disorders or worker performance or work productivity; few have examined work disability	Employment after liver transplantation: a review [86]
Cancer and work model [25]	This evidence-based model includes work environment and demands factors, as well as function and health variables. Four outcomes are addressed including return to work, work ability, work performance, and sustainability (retention)	Predictors of employment among cancer survivors after medical rehabilitation: a prospective study [87]

## Methodological Strengths and Weaknesses of Workplace Factors Research

Strengths in the research methods used to date include the identification of a number of important workplace factors across many diseases/disorders. Stronger observational epidemiological research designs are being used more frequently with a progression from mainly cross-sectional studies to more prospective cohort study designs. Large administrative databases have been used to understand information on a limited number of workplace factors from these sources to enhance statistical power.

Weaknesses are varied and include important methodological concepts. First, the sampling procedures used in most studies of workplace factors are limited. Workplaces are often selected for study through existing researcher networks. There are few studies that use a random sample of workplaces selected for study. This provides the opportunity for participation/selection bias. Second, most studies have been conducted on large workplaces. This is important for purposes of statistical power, but limits the generalizability of findings to medium or smaller enterprises. Third, limited methodologies have been used for analysis. Logistic regression, Cox proportional hazards modeling, and other forms of regression analysis are common. An understanding of the interactions between the worker and the workplace is lacking. There have been few studies using structural equation modeling that can examine modifiers and mediators in a path analysis. Fourth, workplace factors are often measured as perceptions from either the worker or employer. An integrated approach in which both the worker perspective and the organisational perspective are combined would be of added value for our understanding of workplace factors. Fifth, we need more insight into how workplace factors influence work disability (i.e., an understanding of the “etiologic mechanism”). Intervention studies including process evaluations can be helpful to understand this “etiologic mechanism”.

## The Employer Perspective: The Grey Literature

To provide a comparative view of workplace factors from the employer perspective, we reviewed 33 employer-directed publications (“grey literature”) made available by the conference organizers. These articles were a heterogeneous collection of documents summarizing expert and legal opinions, case studies, success stories, management surveys, and best practice guides intended for an employer (and sometimes policy-maker) audience and primarily focused on organizational efforts to manage, prevent, or accommodate disability at work. Authors and publishers of these documents included large employers, vendors, consultants, insurers, regulatory and governmental authorities, employer consortiums, public policy institutes, and charitable organizations. All documents were freely available in English language and published in North America, Europe, or Australia/New Zealand.

For the most part, the “workplace factors” described in the grey literature consisted of organizational policies and practices, but other workplace and workforce characteristics (e.g., aging workforce, regulatory environment, labor union representation, etc.) were sometimes mentioned, typically as background or contextual issues. Most were action-oriented and provided a strong business rationale along with specific “how to” steps necessary for organizational implementation. Systematic empirical support was cited in some, but not all, publications; instead, case study results and expert opinions were more typical. Some employer recommendations were similar across jurisdictions, but others reflected important differences in laws and disability systems, often paralleling the different geographic areas. Large employers, with more staffing and vendor resources, were generally both the initiators and targets of recommendations, with very little content explicitly directed to small- or medium-sized businesses, which may limit the generalizability of the experiences and recommendations made in these articles.

From the 33 grey literature articles, key messages and terminology were extracted, tabled, and categorized to provide a summary of workplace factors commonly addressed by employers with regard to disability management. These were organized into 12 key domains shown in Table 2.

**Table 2:** Summary of 12 workplace factors drawn from a sampling of disability-related employer publications

Key domain	Subtopics and descriptors
(1) Senior management buy-in, commitment, and funding support	Established risk reduction goals DM training for senior managers Visible management commitment Supportive work environment Support and funding from top-down Established leadership in DM practices Health is a part of productivity goals Financial support for DM program is available Company culture is acknowledged as a factor
(2) Clear written policies, guidelines, and procedures	Have an official guideline document Involvement of multi-stakeholder team to develop Established DM eligibility and duration Integration with existing structures Integration of DM with absence management Embrace non-discrimination Have a formal RTW policy Communication of clear objectives
(3) Identifiable RTW coordinator with accountability and suitable training	Designated single RTW coordinator Training and support Guidance committee Strategic plan for RTW coordinator Built infrastructure to support RTW Centralized funding for RTW support Ensured effective management support Established shared accountability
(4) Development and use of practical tools, documents, materials, and consultant reports	Employee packets, educational materials Standard job analysis documents Ergonomic assessments Clear, easy to use information Catalog of accommodations Training, manuals, and courses More effective use of job descriptions
(5) Routine, but individualized, job modification efforts	Policy of routine offer of modified duty No disadvantage to co-workers No disadvantage to supervisor Deal with individual differences Address mobility and accessibility Listing of transitional duties User-friendly accommodation Centralized budget for accommodations More meaningful limited duty work
(6) Training and education of frontline supervisors and disability management staff	Increased breadth of supervisor role DM training for supervisors Awareness of supervisors Consistency among supervisors Accountability of supervisors Involvement of supervisors in RTW planning
(7) General workforce education, outreach, surveillance, and health messaging	Availability of description of procedures in employee handbook Intra-company communications about DM Availability of risk screening for long term disability risk Availability of behavioral health assessments Programs for workers with chronic disorders Supportive organizational culture overall Health-enhancing work environment Employee brochures detailing DM program Enhancement of job retention with declining health Managers as role models for healthy lifestyle
(8) Proactive case management and early RTW planning	Regular case reviews RTW planning in parallel with treatment Establishment of urgency of RTW efforts Avoidance of delays in reporting illness Immediate start absence management Early SAW planning for chronic disorders
(9) Effective use and engagement of medical providers and vendors	Availability of on-site clinics and therapies Incentives to providers for RTW Inform providers of workplace demands Increased communication with provider Utilization of EAP, wellness, and behavioral health Hire providers with employment focus Increased control of sick notes Expert advice for job accommodations Physicians should be educated about guidelines Investment in disease management programs
(10) Involvement, communication, and collaboration with affected workers	Worker awareness of RTW program Involvement of workers in RTW planning Positive perceptions about RTW Early and considerate contact with worker Social and workplace realities Trust and confidentiality Mental health and job stress Tailoring to individual needs Empathy and willingness to help Transparency of process Involvement of employee input
(11) Monitoring of sickness and disability outcomes	Monitor RTW outcomes of programs Case documentation Tracking of cost savings from new programs Analyses of data of RTW outcomes Sickness monitoring
(12) Taking into account workforce and job characteristics	Worker motivation and readiness Traumatic vs. progressive injury or illness History of previous periods of disability Gender and age Attitude of co-workers Excessive or ineffective treatment history Family lifestyle and culture Job tenure, experience, and training Essential elements of the job Difficult or complex cases Extent of medical restrictions Degree of impairment

*RTW* return-to-work, *DM* disability management, *SAW* stay-at-work, *EAP* employee assistance programs

### Defined Roles and Responsibilities

Several domains focused on the roles and responsibilities of specific individuals within the organization’s management umbrella. For example, the buy-in, commitment, and funding support of senior management were described as a necessary precursor to an effective DM strategy. This included both tangible management support (i.e., funding and delegation of responsibilities) and more general aspects of communication that endorsed DM policies within the spirit of supporting employee wellness, non-discrimination, and job retention. The role of frontline supervisors was also mentioned as an important factor, with more effective DM organizations granting frontline supervisors more autonomy, training, and support to improve the consistency and accountability of job modification efforts. Identification and training of an in-house RTW coordinator or disability manager was another key factor.

The effectiveness of medical providers and vendors to facilitate RTW and support job modifications was also seen as an area within the employer’s sphere of influence, especially in jurisdictions where large employers contract directly for private health insurance, occupational health services, disability case management, and employee assistance program (EAP) vendors. Educating or selecting these providers to be occupationally focused and aware of physical job demands and organizational constraints was a relevant workplace disability factor within at least some level of employer control. Related recommendations were to consider the use of on-site clinics and therapies, to increase communication with providers around issues of job modification and RTW, to have meaningful and valid job descriptions, and to offer financial incentives to providers tied to disability performance measures.

### Available Tools and Procedures

Other workplace disability factors pertained to the use of specific tools and procedures. Using administrative data to regularly monitor, evaluate and analyze disability outcomes and trends was considered a useful practice. Evidence of clear, written DM guidelines was a key workplace factor, and these guidelines were likely to be more effective if developed in collaboration with a multi-stakeholder team including disabled or affected workers. Having these guidelines well integrated with other corporate structures and guidelines (e.g., sick leave policy, worksite health promotion, anti-discrimination policies) was also suggested to improve disability outcomes. Other specific tools and resources included ergonomic assessments, generating a customized catalog of possible accommodations, and designing and distributing employee educational packets.

### Prompt and Proactive Response

In addition to the identification of specific roles and resources, some workplace factors pertained to the collective organizational response to disability issues more generally. These included routine offers of job modification and accommodation, general workforce education and outreach to publicize benefits and policies, and early and proactive RTW planning in parallel with medical treatment and rehabilitation. Job modification efforts were viewed as more effective if tailored to individual specifications, if modified duties were purposeful and non-pejorative, and if care was taken not to disadvantage co-workers and supervisors. Promptness and proactive communication were viewed as critical elements of successful job accommodation and RTW.

### Attention to Individual Needs and Circumstances

Another set of workplace factors focused on involvement and collaboration with the affected worker, and the need to consider individual, group, and job characteristics that might alter RTW recommendations or accommodations. In particular, these publications recognized social and behavioral influences that might vary by case and the need to establish sufficient trust and rapport as employers address sensitive issues around health and function at work. In addition to the nature and extent of health impairment, a number of other worker and workplace characteristics were identified; for example, family lifestyle and culture including issues of work/family conflict, job tenure and experience, worker motivation and readiness, prior disability absences, negative preconceptions about workers’ compensation and other regulatory and benefit structures, ineffective treatment history, and the identification of essential elements of the job. From the perspective of employers, understanding these individual and job characteristics in the context of disability was a critical, but sometimes uneasy or complex process.

## Models Underlying Employer Decision-Making

While the grey literature articles did not name specific theoretical or organizational decision-making models, the rationale and explanations suggested several motivational influences, and we labeled these organizational frameworks as the *biomedical model*, the *financial management model*, the *personnel management model*, and the *organizational development model*. Characteristics of the four models and their potential implications for disability management practices are shown in Table 3. For a typical company, decision-making about health and disability issues would involve simultaneous application of these four models, reflecting the company’s multiple roles and responsibilities.

**Table 3:** Four models describing aspects of employer-level decision-making regarding disability management practices

Model	Core rationale or motivation	Decision-making criteria	Primary responsibility for RTW	Intended consequences	Unintended consequences
Biomedical model	Disability of workers is a private, medical concern	Provider judgments of suitability for work	Health care providers	DM programs and decisions are left to experienced and knowledgeable professionals	Providers may lack workplace details; workers feel ignored or forgotten; minimal workplace problem solving and support
Financial management model	Disability of workers consumes valuable company assets	Lost-time costs; Cost of services and vendors	Health care providers	DM programs and decisions are streamlined and designed to reduce short-term costs	Contribute to poor labor-management relations; Higher long-term disability and health care costs
Personnel management model	Disability of workers requires attention to legal requirements	Adherence to laws, regulations, and insurance and benefit plans	Human resources and benefits departments	DM programs and decisions are fair and consistent, with good documentation to defend against legal challenges	Inability to solve complex cases or establish trust and rapport with affected workers
Organizational development model	Disability of workers can be mitigated or prevented by workplace support and communication	Conformance with corporate health and wellness culture	Distributed responsibility between workers, supervisors, managers, and Human Resources staff.	DM programs are more proactive and integrate individual preferences and characteristics of working groups	Higher short-term cost; Greater need for organizational commitment and investment in internal DM resources

*DM* disability management

### Biomedical Model

The organization is made up of workers who are susceptible to injury or illness, but the responsibility for absence management and disability determination should reside with medical professionals. In terms of disability management, this is the perspective that depends on effective use of vendors and consultants for RTW case management and for determining suitability for work. Using this framework, optimal disability management strategies are those that access the most effective medical and case management teams outside of the company. Potential implications for disability management are reduced employer support and assistance, a higher burden and expectation placed on medical providers, and greater potential for workers to feel ignored or unsupported.

### Financial Management Model

The organization is a corporate entity with responsibility for making prudent financial decisions. In terms of disability management, this is the perspective that relates to financial decision-making, cost-containment, bottom line, benefit-cost ratios, disability cost outcomes, and monitoring of statistical trends. Using this framework, optimal disability management strategies are those that carry the least cost, financial liability, and staffing burden. Though lower cost options might be appealing on the surface, negative implications for disability management are the absence of ancillary services or professional linkages to facilitate RTW, workers reluctant to RTW due to poor labor-management relations, and short-term financial gains made at the expense of long-term health and disability costs.

### Personnel Management Model

The organization has a regulatory and fiduciary responsibility to treat employees’ concerns promptly, fairly, and consistent with best practices and regulatory guidelines. In terms of disability management, this is the perspective that relates to establishing clear written guidelines, training and accountability of managers and supervisors, incident tracking and case management, early RTW programs, better communication with other stakeholders, adherence to applicable laws and standards, dealing with workforce problems, and effective management of employee benefits. Using this framework, optimal disability management strategies are those that are responsive (but do not necessarily exceed) all applicable regulations and best practice standards. Potential implications for disability management are efficient and seamless communication, fair administration of benefits, and proactive tracking and support; however, unusual cases or delayed absences may be poorly understood or lack opportunities for a more individualized approach.

### Organizational Development Model

The organization has a unique identity and culture that influence job satisfaction and productivity, competitiveness, and innovation. In terms of disability management, this is the perspective that relates to workforce outreach and support, collaboration and problem solving with affected workers, overt management support for wellness and safety, leadership development, and more individualized job modification efforts. Using this framework, optimal disability management strategies are those that are emblematic of the company’s overall culture of diversity, wellness, inclusivity, labor-management relations, and sensitivity to the needs of workers. Potential implications for disability management are more individualized and collaborative efforts to solve disability problems, but this may come at a higher financial cost and require a consistently high level of organizational commitment to employee health and well-being.

## Input from the Special Panel and Conference Attendees

In general, the special panel reinforced the workplace factors we found in the grey or scientific literature. Factors emphasized by the panel included employer ability/willingness to accommodate, job satisfaction/employee engagement, a psychologically safe workplace, physical safety and job demands, leadership, and supervisor beliefs. Attendees reiterated the importance of employer buy into the proposed research—this is vital for research on workplace factors. There was one workplace factor that was brought to our attention that was not captured elsewhere, and that was a misalignment of hierarchy in leadership. Decisions related to workplace disability management are not necessarily based on evidence, but on preference of the management. This important aspect is generally neglected in research as it is difficult to capture in the daily processes related to work disability prevention.

## Disparities Between Research Factors and Employer Areas of Concern

Identifying disparities between workplace factors researchers have considered and areas employers are concerned about may lead to the identification of avenues for potential research. Three of these incongruences include perspective, outcomes, and type of disability focus.

The grey literature takes a managerial perspective, whereas the research literature tends to focus on the individual worker. This distinction is pervasive throughout conceptual frameworks. Of the twelve domains of workplace factors identified in the grey literature (Table 2), only two come close to taking the worker perspective (#10 on involvement and #12 on workforce & job characteristics). However, even in the case of #10, the focus is on how management can better involve and communicate with workers, and thus the focus still comes back to management. Of the four conceptual models implied in the grey literature, only one (the organizational development model) takes into account the worker’s individualized needs and reactions to disability management programs. This is also arguably the least common perspective taken in the grey literature. In the research literature, there is discussion of work organization and support factors in the conceptual frameworks, but these are typically focused on how workers perceive management’s efforts, rather than having a managerial perspective (e.g., what specific policies and programs are most effective). As a result, the models used in the research literature do not include the level of specificity found in the grey literature with regard to disability management policies, procedures, and systems.

The outcomes implied in the grey literature and research literature conceptual models differ. In general, the grey literature reflects the executive subculture [59], which tends to be financially focused, depersonalized, systems-focused, and generally removed from the experiences of the line worker. As a result, the conceptual models tend to emphasize putting systems into place that are financially viable and that will increase productivity with limited cost. The interest is in overall rates and financial numbers, rather than specific individual cases. Even in the most “worker-friendly” model, the organizational development model, the focus tends to be on creating an overall culture in the organization rather than issues specific to individual workers. In contrast, the research model is focused on the individual’s outcomes and what leads to an individual’s return to work, with a heavy emphasis on the individual’s characteristics, behavior, stress levels, and attitudes (e.g., satisfaction). From a level of analysis perspective, the outcome is at the organizational level in the grey literature whereas the outcome is at the individual level in the research literature.

The grey literature rarely mentions individual disabilities and is more focused on general disability management policies, whereas the research literature is more likely to differentiate types of disabilities and the return to work issues specific to certain disabilities. The focus on general policies in the grey literature is in line with the managerial perspective and emphasis on the organizational level of analysis in that literature. Issues associated with specific disabilities are more likely to be viewed as inefficiencies in the system; the goal is to have consistency in the system and to maximize the positive outcomes across all disabled workers, rather than for individual workers with specific disabilities. The research literature emphasizes the individual’s experience, and thus it is a natural extension to consider how the factors impacting return to work vary for different individuals, particularly in terms of the type of disability. Although the research literature has begun to develop more models that cut across multiple disabilities, the core assumption is that there will be some commonality across disabilities but also some specific issues related to each type of disability, as opposed to the grey literature which only considers issues generalizable to all disabilities.

## Implied or Actual Theoretical Perspectives Guiding Research and Practice

Research and intervention related to the domain of workplace factors associated with disability has proceeded in the absence of a unified conceptual framework. While a number of models have been put forward, or are implied in the nature of research or interventions that have been initiated, none appear to have played a significant role in prompting research, or as a lens to guide study questions or the interpretation of findings.

One feature that appears to have impeded development and uptake of a conceptual framework of workplace factors related to disability concerns the nature of variables that we constitute the basic units of analysis of the domain. The present review lists four broad classes of workplace factors related to disability, (1) physical jobs demands, (2) psychosocial job demands, (3) work organization and support, and (4) workplace beliefs and attitudes. These factors are difficult to combine meaningfully into a theoretical framework because they vary according to the degree to which they can be defined and assessed independent of characteristics the worker. Of the four workplace factors listed above, only the first and third, physical job demands and work organization, can be assessed independent of characteristics of the worker. Factors such as job strain, fairness or job satisfaction can only be assessed by examining the worker’s ‘perception’. Unless findings within workplaces showed a high degree of consensus in the manner in which workers respond to questions about job strain, fairness or job satisfaction, these factors might best be construed as worker characteristics as opposed to workplace characteristics. Without some effort to bring greater definitional clarity to the units of analysis relevant to a domain of enquiry, it is unlikely that a viable conceptual framework will emerge to guide research or intervention.

**Fig. 1:**
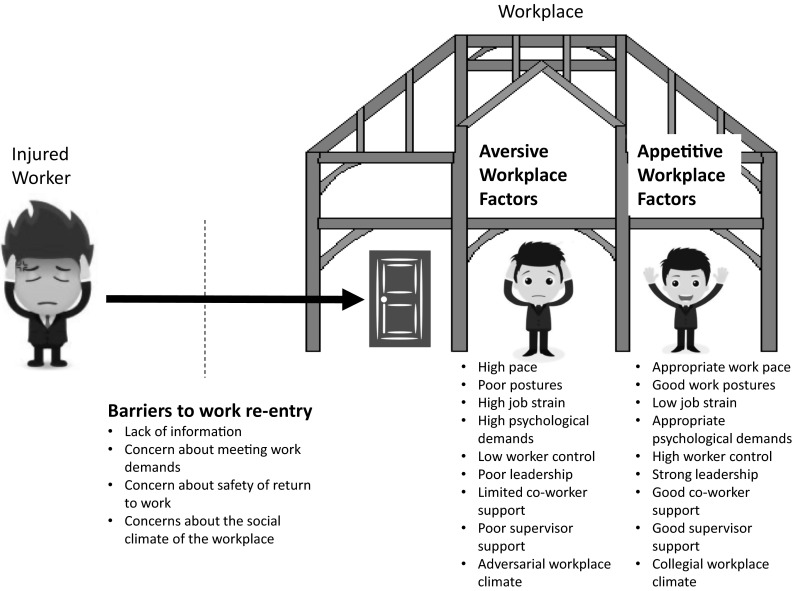
Three basic principles for guiding research and practice showing common workplace factors

In any complex area, such as work disability, there can be advantages to starting with only the most basic principles as the building blocks of a conceptual framework. Three basic principles are required for the development of a conceptual framework necessary to guide research and intervention in a meaningful way: (1) barriers to work re-entry, (2) aversive factors in the work environment, and (3) the appetitive value of the work environment (Fig. 1).

### Barriers to Work Re-Entry

If we assume that the injured worker has return-to-work as a primary goal, then we know that the injured worker will want to strive toward achieving this goal. Since not all injured workers return to work, it follows that there might exist important barriers to work re-entry. From a workplace perspective, it then becomes important to identify all the barriers that a motivated injured worker might face in efforts to return to work. The injured worker might lack information about how to proceed, the injured worker might have concerns about his/her ability to effectively meet the demands of employment, the individual might have inaccurate information about the safety of returning to work, the injured worker might have concerns about the social climate of work re-entry.

The research questions emerging from this perspective would include identification of all possible work re-entry barriers and examination of the worker characteristics that might moderate the impact of the workplace factors. To the degree that these factors are shown to account for significant variance in disability outcomes, intervention strategies targeting the workplace or the worker could be initiated.

### Aversive Factors in the Workplace

A basic tenet of human nature is that individuals avoid environments or situations that are experienced as aversive. Workplaces are environments that can be graded according to their aversiveness. The aversive characteristics of the workplace might be related to physical elements of the work environment (e.g., noise, temperature, pace, smell), or social/interpersonal elements (e.g., disrespect, aggression). The greater the number of aversive characteristics of the work environment, the more likely that the injured worker will be motivated to avoid returning to work.

The research questions emerging from this perspective would include identification of all physical and interpersonal aversive characteristics of a work environment as well as the worker characteristics that might moderate the impact of these aversive characteristics. To the degree that these factors are shown to account for significant variance in disability outcomes, intervention strategies targeting the workplace or the worker could be initiated.

### The Appetitive Value of the Workplace

A basic tenet of human nature is that individuals are drawn toward environments or situations that are experienced as positive. Workplaces are environments that can be graded according to their appetitive value. Appetitive (or positive) characteristics of the workplace might include physical elements (e.g., comfort, flexibility, financial reward), or social/interpersonal elements (e.g., social contact, identity, autonomy, control). The greater the number of appetitive characteristics of the work environment, the more likely that the injured worker will be motivated to return to that environment.

The research questions emerging from this perspective would include identification of all physical and interpersonal appetitive characteristics of a work environment as well as the worker characteristics that might moderate the impact of these appetitive characteristics. To the degree that these factors are shown to account for significant variance in disability outcomes, intervention strategies targeting the workplace or the worker could be initiated. A conceptual framework emerging out of these three basic principles, namely barriers, aversive factors, and appetitive value, could provide a useful foundation for assessment and intervention aimed at reducing work disability. One might envisage a set of assessment procedures that would yield a graded profile of a particular workplace along dimensions of barriers, aversive factors, and appetitive value. Relative strengths and weaknesses revealed through such a profile could then point to avenues of intervention intended to reduce the degree of work disability associated with a particular workplace.

## Conclusion/Research Recommendations

Based on our review, we have established three broad recommendations for future research in the area of workplace factors and disability prevention: (1) Incorporate more advanced approaches to analysis; (2) Include small and medium sized enterprises; and (3) Consider workplace factors from all relevant domains.

### Incorporate More Advanced Approaches to Data Collection and Analysis

The levels at which workplace factors are appraised within organizations may have an impact on the types of disability prevention strategies that are the product of research. As shown in Table 4, four levels of assessment are apparent from the existing literature: (1) information from the perception of *ill or injured workers*, usually in the form of psychosocial questionnaires, physical task inventories, or semi-structured interviews; (2) information from the *workforce as a whole*, usually in the form of job descriptions, safety climate surveys, or other industry descriptors; (3) assessments of *supervisor attitudes*, e.g., their willingness to implement and support job modifications; and (4) *organizational practices and procedures* as viewed by managers within the organization. Assessment at each level implies a different solution to disability challenges. For example, if individual-level perceptions of demanding and stressful work are the focus of research, then recommended interventions will likely include individual case-level support and problem-solving. If characteristics of the workplace are assessed, then recommended interventions might focus on engaging co-workers and improving workforce awareness. If supervisors are assessed, then interventions will relate to supervisor training and reinforcement. If managers are assessed, then changes to policies and procedures would be the target for organizational change.

**Table 4:** The significance of appraising workplace factors at different levels within organizations

Examples of workplace factors assessed at this level	Implied nature of disability problems	Most appropriate type of intervention strategy
Worker level		
Worker perceptions of psychosocial job demands (lack of control, role ambiguity, job stress, unfairness)	Workers who report more stressful jobs feel less able to manage symptoms and control workload to prevent disability	Provide individual-level stress management and methods to improve personal control
Worker perceptions of physical job demands (fast pace, heavy work ratings, awkward posture)	Workers who rate their jobs as more physical have fears about pain escalation or re-injury.	Focus on job demands of greatest concern to individual workers
Workforce level		
Co-worker support	Preventing disability sometimes requires coordination and support of co-workers	Include affected co-workers in plans for job accommodation or return-to-work
Health and safety climate	Disability prevention may be incongruent with the shared values of workers in a particular line of work	Provide general workforce re-education and improve awareness
Supervisor level		
Support for job modifications	Disability prevention efforts may fail without adequate supervisor support for job modifications	Train supervisors to translate medical restrictions into job modifications and facilitate needed accommodations
Communication and follow-up	Disability prevention requires positive communication and regular support with the affected worker	Train supervisors to take a larger role in supportive communication with ill or injured workers
Managerial level		
Proactive return-to-work policies and practices	Organizations may fail to provide the procedural infrastructure for solving disability problems	Disability prevention should be based on a clear set of policies and procedures that are uniformly applied in individual cases
Managerial commitment to worksite safety and employee health and wellness	Organizations fail to communicate messages of employee concern and empathy needed to prevent disability	Disability prevention should be part of a broader campaign to support employee health and wellness at the highest levels

The choice of researchers to assess various levels within organizations reflects, to some degree, their implicit beliefs about the underlying causes of unnecessary sickness absence and work disability. A focus on workers implies that disability outcomes are mediated by individual worker beliefs and perceptions. A focus on the workforce as a whole suggests that commonly-held attitudes and beliefs within the organization play a role in disability outcomes. A focus on supervisors implies that disability prevention efforts are not sufficiently coordinated and supported at the working group level. A focus on managers implies that the basic organizational climate is not supportive of disability prevention efforts. To clearly understand workplace factors that influence disability, future research should strive for multi-level assessment that includes attention to all four levels, thus providing a more complex view of the problem from a variety of perspectives. In reality, however, conducting such an extensive assessment of disability-related factors within an organization requires a high level of trust with the host organization.

### Include Small and Medium Sized Enterprises

Most research on workplace factors has been conducted in large workplaces. Many of these workplaces have established partnerships with researchers and continue to be a source population of workers and workplace factors for study. These large workplaces may have resources not available at smaller companies. Therefore, the generalizability of these research findings to smaller and medium-sized employers becomes questionable. Future research needs to explore ways in which we can include small and medium-sized employers (which may require different research designs) and also strive for better understanding of companies who do not participate in studies of workplace factors (potential selection bias).

### Consider Workplace Factors from All Relevant Domains

Many studies of workplace factors have recognized and assessed the variability in workplace physical demands and working style, but may have neglected supervisory and working group support. Future research should attempt to incorporate support variables, corporate policies and practices, and physical demand variables to assess their relative contributions to work disability. A focus on factors that represent modifiable targets may be helpful for intervention identification or development, but also may miss important subgroups where the intervention could be more or less effective. We recommend a balanced approach when considering workplace factors that assumes shared responsibilities for disability prevention between the employee and employer.

Therefore, necessary next steps in workplace factors research include the incorporation of information from all organizational levels within the workplace, the inclusion of small and medium-sized employers, and a comprehensive assessment of variables from all domains in the workplace. Major obstacles for achieving these steps include gaining employer support to conduct such comprehensive examinations of the workplace, and the lack of study design for quantitative assessment of small and medium-sized employers. In order to assess factors from all workplace domains and levels, a workplace will need to be very cooperative and accommodating. In addition, internal communication is very important as information about the research project should not only be available at the management level, but also at departmental and individual worker levels. This requires a broader communication approach in which effective company channels are indispensable. Given most workplaces focus on production or delivery of services, accommodating such an intrusive measurement exercise may be too burdensome. Researchers should find new ways to work with employers to obtain the necessary measures. Further, to incorporate data from small and medium-sized enterprises, researchers will need to be creative in their approach to data collection and analysis. Different research designs may be needed that combine small data samples into one analysis to obtain meaningful results. This also requires a change in the research community mindset, where large scale quantitative research is regarded as more robust compared to other designs such as qualitative, participatory or action research designs. Tackling these hurdles will improve future research on employer disability prevention strategies.
